# Harmonization of [^11^C]raclopride brain PET images from the HR+ and HRRT: method development and validation in human subjects

**DOI:** 10.1186/s40658-022-00457-z

**Published:** 2022-04-13

**Authors:** Jocelyn Hoye, Takuya Toyonaga, Yasmin Zakiniaeiz, Gelsina Stanley, Michelle Hampson, Evan D. Morris

**Affiliations:** 1 Department of Radiology and Biomedical Imaging, Yale School of Medicine, New Haven, CT USA; 2 Department of Psychiatry, Yale School of Medicine, New Haven, CT USA; 3 Yale Positron Emission Tomography (PET) Center, Yale School of Medicine, New Haven, CT USA; 4 Department of Biomedical Engineering, Yale University, New Haven, CT USA

**Keywords:** Harmonization, Brain PET, Binding Potential, [^11^C]Raclopride, Inter-scanner, Test–retest Variability

## Abstract

**Background:**

There has been an ongoing need to compare and combine the results of new PET imaging studies conducted with [^11^C]raclopride with older data. This typically means harmonizing data across different scanners. Previous harmonization studies have utilized either phantoms or human subjects, but the use of both phantoms and humans in one harmonization study is not common. The purpose herein was (1) to use phantom images to develop an inter-scanner harmonization technique and (2) to test the harmonization technique in human subjects.

**Methods:**

To develop the harmonization technique (Experiment 1), the Iida brain phantom was filled with F-18 solution and scanned on the two scanners in question (HRRT, HR+, Siemens/CTI). Phantom images were used to determine the optimal isotropic Gaussian filter to harmonize HRRT and HR+ images. To evaluate the harmonization on human images (Experiment 2), inter-scanner variability was calculated using [^11^C]raclopride scans of 3 human subjects on both the HRRT and HR+ using percent difference (PD) in striatal non-displaceable binding potential (BP_ND)_ between HR+ and HRRT (with and without Gaussian smoothing). Finally, (Experiment 3), PD_T/RT_ was calculated for test–retest (T/RT) variability of striatal BP_ND_ for 8 human subjects scanned twice on the HR+.

**Results:**

Experiment 1 identified the optimal filter as a Gaussian with a 4.5 mm FWHM. Experiment 2 resulted in 13.9% PD for unfiltered HRRT and 3.71% for HRRT filtered with 4.5 mm. Experiment 3 yielded 5.24% PD_T/RT_ for HR+.

**Conclusions:**

The PD results show that the variability of harmonized HRRT is less than the T/RT variability of the HR+. The harmonization technique makes it possible for BP_ND_ estimates from the HRRT to be compared to (and/or combined with) those from the HR+ without adding to overall variability. Our approach is applicable to all pairs of scanners still in service.

**Supplementary Information:**

The online version contains supplementary material available at 10.1186/s40658-022-00457-z.

## Background

Quantitative dynamic PET neuroimaging can be used to calculate estimates of receptor availability in the brain via model parameters such as non-displaceable binding potential (BP_ND_). However, BP_ND_ estimates may not be consistent from one scanner to another if scanner resolutions differ. PET images with poorer spatial resolution exhibit more spill-out/spill-in, which can lead to under/overestimation of BP_ND_, particularly for small regions of interest with high contrast [1, 2]. Given the influence of resolution on BP_ND_ estimates, there is a need to harmonize PET images before estimating and comparing or combining BP_ND_ values from images obtained on different scanners.

Previous studies have developed inter-scanner harmonization techniques for PET images using only *phantoms* [3, 4] or only *human subjects* [5, 6]. Makris et al. [3] evaluated strategies for harmonizing FDG PET/CT data for estimation of SUV in multicenter trials using *phantoms*. Specific to neuroimaging, Joshi et al. [4] described a method to smooth FDG-PET images from fifteen different scanners (for the multicenter Alzheimer's Disease Neuroimaging Initiative (ADNI) study) to a common resolution using scans of a 3D Hoffman Brain *phantom*. Scans of the 3D Hoffman brain phantom were smoothed with the necessary Gaussian smoothing such that all scanners would have a comparable resolution of 8 mm full width at half maximum (FWHM) (in-plane and axial). Van Velden et al. [5] harmonized [^11^C]flumazenil *human subject* images on both the HR+ and the HRRT by blurring the HRRT images with a 6 mm FWHM Gaussian filter to match the resolution of the HR+ scanner. In another neuroimaging study, van Velden et al. [6] scanned *human subjects* using [^11^C]verapamil, [^11^C]raclopride, and [^11^C]flumazenil on both the HRRT and HR+ scanners by blurring HRRT images with a 6.5 mm FWHM Gaussian filter. Given that prior studies have been either phantom-based or human subjects-based, we felt it would be illustrative to go one step further by first developing harmonization techniques in phantoms on the scanners of interest (in this case, the HR+ and HRRT) and then validating those techniques in human subjects.

Also motivating the present study was our need to compare newly acquired striatal BP_ND_ estimates from HRRT images with a previously published population of striatal BP_ND_ estimates from HR+ images [7]. Here, we used phantom images to develop a harmonization method by blurring HRRT images to the resolution of HR+ images with a 3D Gaussian filter. We validated the approach by applying it to images from three human subjects scanned on both scanners.

## Methods

### Experiment 1: Harmonization development

#### PET scans

The Iida brain phantom [8] was filled with F-18 FDG. F-18 FDG was mixed well with 800 ml water to create a uniform concentration solution. The mixture was poured into the phantom with an injected activity of 2.9 mCi. Once the phantom was full, it was capped and shaken to move the air bubbles to the top. Then, the cap was reopened to top off the phantom with additional F-18 FDG solution. This procedure was repeated several times to minimize air bubbles. The phantom was imaged on a single day, sequentially, on a series of PET scanners including the ECAT HR+ (HR+; Siemen’s/CTI) and the High-Resolution Research Tomograph (HRRT; Siemens/CTI). The phantom was gently moved between scanners to avoid disturbing any residual air bubbles. Static images of the phantom were acquired for 15 min each, first on the HRRT and then on the HR+. Because of decay, the radioactivity concentration in the phantom was 70.22 kBq/mL at the start of the HRRT scan and 37.83 kBq/mL at the start of the HR+ scan.

PET emission data were acquired in 3D and corrected for decay, attenuation, scatter, dead time, detector sensitivity, and randoms. The HRRT data were reconstructed with ordered subset expectation maximum (OSEM) using two iterations and thirty subsets at a voxel size of 1.2 mm × 1.2 mm × 1.2 mm and dimensions of 256 × 256 × 207 voxels. The HR+ data were reconstructed with OSEM using 4 iterations and 16 subsets at a voxel size of 2.06 mm × 2.06 mm × 2.4 mm and dimensions of 128 × 128 × 63 voxels. The reported resolution of the HRRT ranges from 2.3 to 3.4 mm FWHM [9]; the reported resolution of the HR+ ranges from 4.1 to 7.8 mm FWHM [10].

#### Determination of harmonization factor

The HRRT image voxel size was altered to match the voxel size of the HR+ images by down-sampling using trilinear interpolation (In-house IDL software). The resized image dimensions for the HRRT were 152 × 152 × 105 voxels and were designated as hHRRT(0 mm). Isotropic Gaussian blurring was applied to the hHRRT(0 mm) image with varying values of FWHM (from 0 to 10 mm in increments of 0.5 mm). Each blurred image was designated as hHRRT(xmm) where x indicates the FWHM of the Gaussian.

The hHRRT and HR+ images were loaded into MATLAB (2018b). The HR+ image was padded with zeros in the x-, y-, and z- dimensions resulting in dimensions of 152 × 152 × 105. The HR+ image was rigidly registered to the hHRRT(0 mm).

The intensity of each hHRRT image of the phantom was compared voxel-by-voxel to the corresponding intensity of the registered HR+ image by calculating a voxel-wise difference. The voxel-wise differences were squared and summed for all voxels across the image volume resulting in a sum of squared errors (SSE),

1 $${\text{SSE}} = \mathop \sum \limits_{i = 1}^{N} \left( {y_{{i,{\text{hHRRT}}}} - y_{{i,{\text{HR}} + }} } \right)^{2}$$

where y_i,hHRRT_ is the intensity of the i^th^ voxel in the hHRRT image and y_i,HR+_ is the intensity of the i^th^ voxel in the HR+ image, and N is the total number of image voxels. The FWHM of the Gaussian kernel that minimized the SSE was deemed optimal.

### Experiment 2: Validation in human subjects

#### Subjects

Three healthy subjects (2 men and 1 woman, mean age 33 ± 6 years, mean weight 73.7 ± 7.16 kg, mean BMI 24.8 ± 2.65) were recruited into the study. This study was approved by Yale University’s Human Investigations Committee, Radioactive Drug Research Committee, and Radiation Safety Committee. Informed consent was obtained prior to the performance of any study procedures. Subjects without significant medical issues, without current or recent smoking or nicotine use, and without current psychiatric disorders were recruited.

#### Study design

Each subject received a structural MRI scan on a Trio 3 T MR scanner (Trio and Prisma, Siemens Medical Systems, Erlangen, Germany). Subjects received a total of two 90 min [^11^C]raclopride scans, each acquired on a separate PET scanner. The MRI scan was acquired on a different day from the PET scans and was used for anatomical localization of PET scans. The pair of PET scans occurred on either the same day (1 subject) or on two separate days less than 7 weeks apart (2 subjects). One subject was scanned on the HRRT first, and the other two subjects were scanned on the HR+ first.

#### PET scans

[^11^C]raclopride was synthesized using methods previously described [11]. Before each PET scan, either a 6-min transmission scan (HHRT) or a 9-min transmission scan (HR +) was acquired for attenuation correction. [^11^C]raclopride was administered as a bolus by a computer-controlled pump (Harvard Apparatus, Holliston, MA, USA). The mean raclopride mass per body weight was 0.03 ± 0.004 μg/kg for the HRRT scans and 0.02 ± 0.009 μg/kg for the HR+ scans.

PET emission data were acquired in 3D and corrected for decay, attenuation, scatter, dead time, detector sensitivity, and randoms. For the HRRT images, motion correction was applied using a Polaris Vicra (Northern Digital Inc.) optical hardware-based motion tracking device [12]. For the HR+ images, motion correction was applied using a mutual-information algorithm [13] and the FLIRT software (http://www.fmrib.ox.ac.uk/fsl/flirt). Emission data were collected for 90 min. HRRT and HR+ data were both binned into 27 total time frames of 6 × 0.5 min, 3 × 1 min, 2 × 2 min, and 16 × 5 min. The HRRT and HR+ data were reconstructed with the same method and voxel sizing as Experiment 1.

#### Image analysis

Individual HRRT and HR+ PET images were co-registered to the subject’s own MR image and then nonlinearly registered to a MR template in a common space (MNI space; Bioimage Suite [14]). ROIs were extracted using automated anatomical labeling [15] (AAL) to generate regional time activity curves. The whole striatum ROI was extracted using the AAL atlas. Time activity curves were fitted with the simplified reference tissue model (SRTM) [16] using the cerebellum as a reference region to calculate non-displaceable binding potential (BP_ND_) as the endpoint.

The harmonization method described in Methods in Sect. 1 was applied to the HRRT data. BP_ND_ estimates were made from the hHRRT(4.5 mm) using the same method as described above for the HR+ and HRRT scans.

#### Comparison of calculated endpoints

BP_ND_ values for the striatum were calculated and compared within subjects across the 3 sets of dynamic PET images: HRRT, HR+, and hHRRT(4.5 mm). The comparison metric was the percent difference (PD) between the HRRT/hHRRT(4.5 mm) and the HR+, which was calculated as follows:

2 $${\text{PD}} = 100 \times \frac{{\left| {{\text{BP}}_{{{\text{HRRT}}}} - {\text{BP}}_{{{\text{HR}} + }} } \right|}}{{\left( {\frac{{{\text{BP}}_{{{\text{HRRT}}}} + {\text{BP}}_{{{\text{HR}} + }} }}{2}} \right)}}$$

where BP_HRRT_ is the striatal BP_ND_ for either HRRT or hHRRT(4.5 mm) and BP_HR+_ is the striatal BP_ND_ for HR+.

### Experiment 3: Comparison of calculated endpoints with Test–Retest Data from HR+

Test–retest (T/RT) measurements of striatal BP_ND_ were calculated using data from a previous study by our group [7]. In that study, eight healthy subjects (four women and four men, mean age 31.3 ± 11.2 years, mean weight 68.7 ± 12.8 kg, mean BMI 23.9 ± 3.31) without significant medical issues were recruited. Current or recent smoking or other nicotine use or personal or family history of psychiatric disorders (including substance misuse) were exclusionary. [^11^C]raclopride scans were acquired on two separate days on the same subjects using only the HR+ scanner. The HR+ data were reconstructed using the same reconstruction method and voxel size as described above. PD_T/RT_ was calculated between the two HR+ striatal BP_ND_ values as follows,

3 $${\text{PD}}_{T/RT} = 100 \times \frac{{\left| {{\text{BP}}_{{{\text{HR}} + ,1}} - {\text{BP}}_{{{\text{HR}} + ,2}} } \right|}}{{\left( {\frac{{{\text{BP}}_{{{\text{HR}} + ,1}} + {\text{BP}}_{{{\text{HR}} + ,2}} }}{2}} \right)}}$$

where $BP_{{{\text{HR}} + ,1}}$ is the BP_ND_ from the first HR+ scan and $BP_{{{\text{HR}} + ,2}}$ is the BP_ND_ from the second HR+ scan.

## Results

### Experiment 1: Harmonization development

**Fig. 1:**
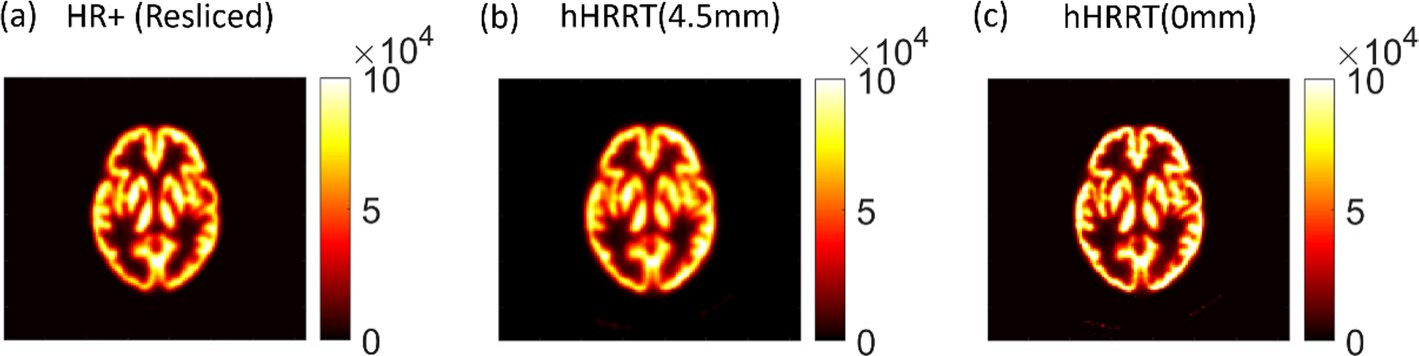
PET images of the Iida phantom filled with F-18 solution **a** HR+ (rotated and translated), **b** the hHRRT(4.5 mm), and **c** hHRRT(0 mm). All hHRRT images were down-sampled to match the HR+ voxel size. Units on the color bars are in Bq/mL

**Fig. 2:**
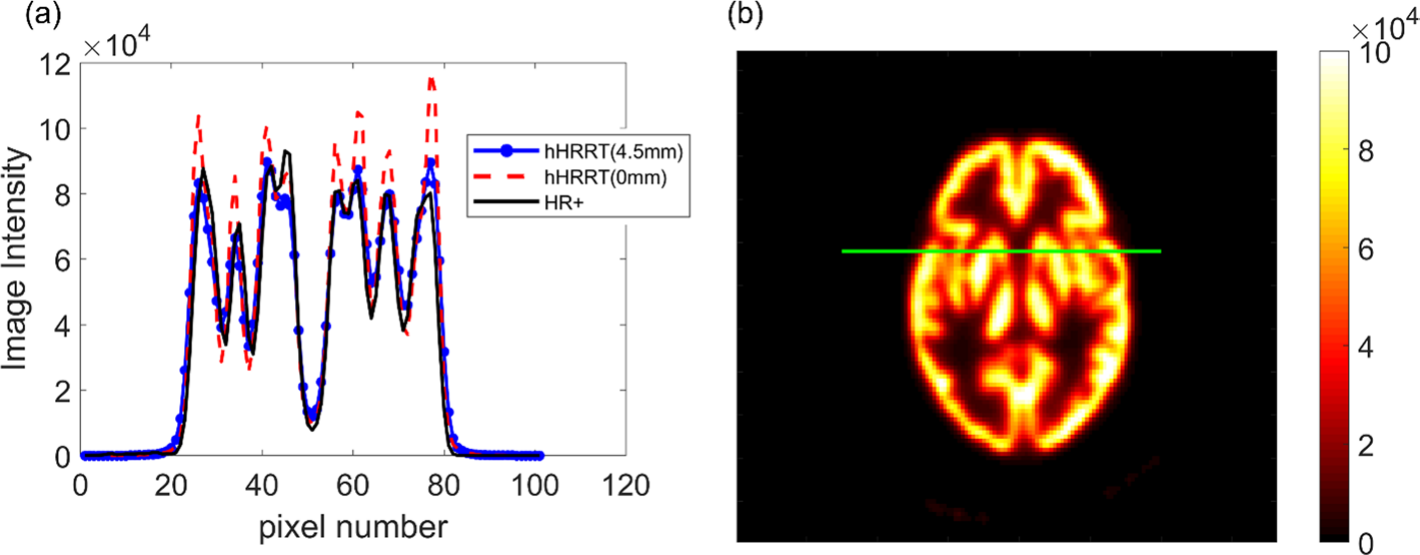
**a** Line profiles through the Iida phantom for the hHRRT(4.5 mm) (blue line), hHRRT(0 mm) (red dashed line), and HR+ (resliced) (black line). **b** An image of the hHRRT(4.5 mm) image showing the line location. The color bar is in units of Bq/mL

The optimal 3D Gaussian kernel was determined to be 4.5 mm FWHM (see Additional file 1: Fig. S1). The hHRRT(0 mm) and HR+ images are shown alongside the hHRRT(4.5 mm) image (Fig. 1). There is good visual agreement between the hHRRT(4.5 mm) image and the HR+ image (Fig. 1). Line profiles (Fig. 2) through the hHRRT(4.5 mm), hHRRT(0 mm), and HR+ images show that the line profile of hHRRT(4.5 mm) was more similar to HR+ than was the line profile of the HRRT to the HR+.

### Experiment 2: Validation in human subjects

**Fig. 3:**
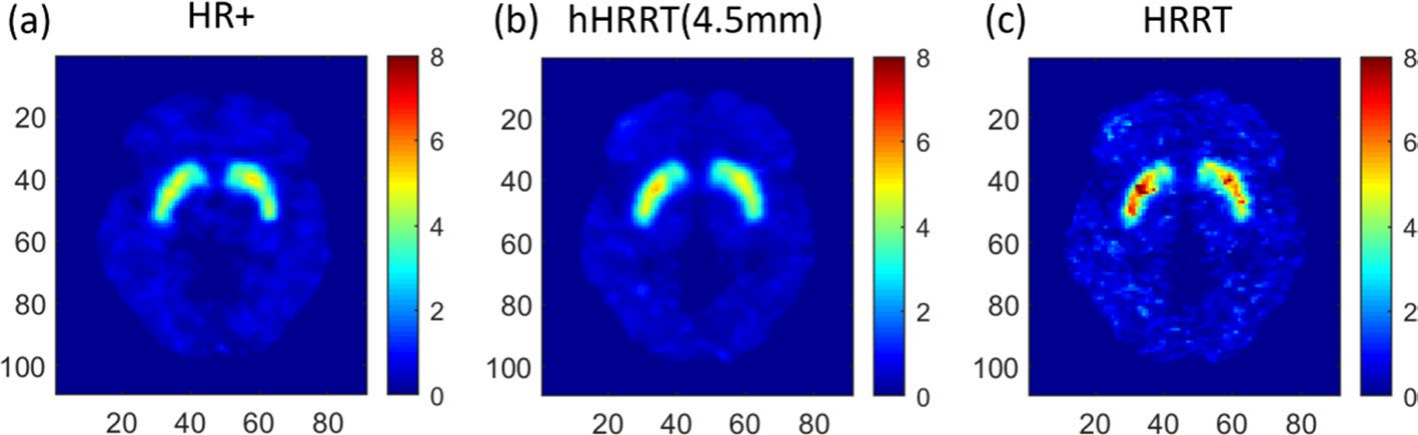
Parametric [^11^C]raclopride BP_ND_ images created using SRTM with cerebellum as a reference region for a single representative human subject resliced in MNI2-space. The images: **a** HR+, **b** hHRRT(4.5 mm), and **c** HRRT. Please note that Fig. 3c is not comparable to 1c because 3c is in native HRRT space before down-sampling. The color bar represents BP_ND_ values which are unitless

**Fig. 4:**
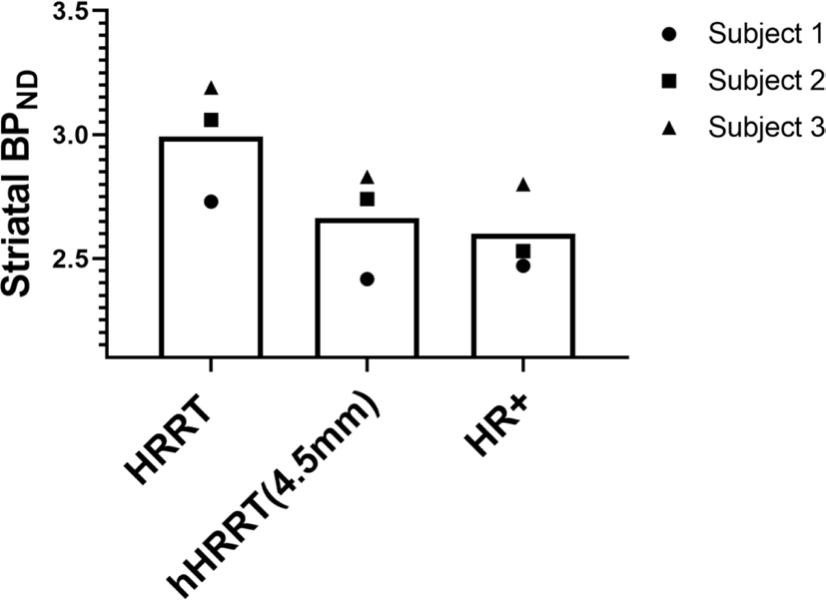
Striatal BP_ND_ for the 3 subjects HRRT, hHRRT(4.5 mm), and the HR+. Subject 1 (filled circle), Subject 2 (filled square), and Subject 3 (filled triangle) are all marked with filled shapes

BP_ND_ images derived from each subject’s hHRRT(4.5 mm) images showed good visual agreement with those generated from HR+ images (Fig. 3), including similar distribution of intensity and resolution. The regional striatal BP_ND_ values for the three subjects were consistently largest for the HRRT (Fig. 4).

### Experiment 3: Comparison of calculated endpoint with Test–Retest Data from HR+

**Fig. 5:**
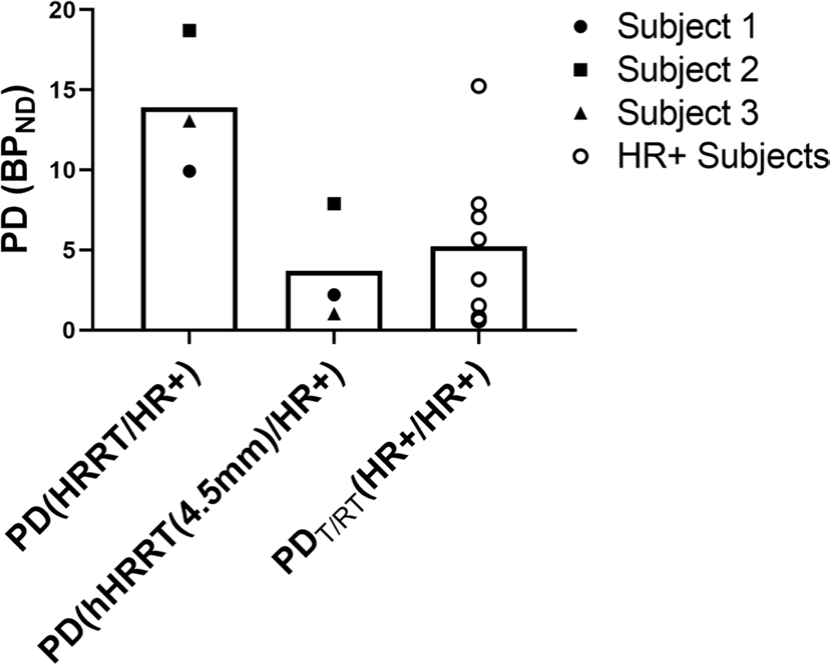
Percent difference in striatal BP_ND_ for the 3 subjects for HRRT and hHRRT (4.5 mm) relative to the HR+ compared with PD_T/RT_ from 8 subjects scanned twice on the HR+. Subject 1 (filled circle), Subject 2 (filled square), and Subject 3 (filled triangle) are all marked with filled shapes. The T/RT subjects are marked with open circles (open circle)

The average PD between HRRT and hHRRT(4.5 mm) images and the HR+ images was 13.9 ± 4.45% for unfiltered HRRT and but only 3.71 ± 3.67% for hHRRT(4.5 mm) (Fig. 5). The mean PD_T/RT_ for 8 subjects scanned twice on the HR+ was 5.24 ± 4.92% (Fig. 5).

## Discussion

Experiment 1 demonstrated that the 4.5 mm FWHM Gaussian filter was optimal for harmonizing HRRT and HR+ [^11^C]raclopride PET images. The validation study in human subjects (Experiment 2) showed that the HRRT striatal BP_ND_ estimates were consistently higher than those from the HR+, and that Gaussian filtering of HRRT images reduced striatal BP_ND_ to be closer to those of the HR+. Experiment 3 showed that the 4.5 mm FWHM Gaussian filter reduced the inter-scanner difference in BP_ND_ to *less* than the mean intra-scanner T/RT variability for the HR+. While there is inherent variability in BP_ND_ estimates from one scan session to another, our findings showed that it is possible to limit the variability between harmonized scans to the average T/RT variability.

Once properly harmonized, BP_ND_ estimates from the HRRT can be combined with those from the HR+, with minimal addition to the overall variance. The ability to combine BP_ND_ data from different scanners could be important in at least three scenarios: (1) Results from one scanner are being compared to and combined with historical published data acquired on a different scanner, (2) in the execution of multicenter trials it may become necessary to harmonize data from different scanners before combining and/or comparing, and (3) a single-site study may be designed to use several different PET scanners to simplify scheduling of patients.

Previous studies have harmonized [^11^C]raclopride PET brain images between HRRT and HR+. Van Velden et al. [6] applied a 6.5 mm FWHM Gaussian filter to HRRT images to harmonize them to HR+ images. The 4.5 mm FWHM found in our study is smaller than theirs, which may be due to differences in voxel sizes and in reconstruction methods used in the two studies. Given that reconstruction methods and voxel sizes can be important for determining harmonization schemes, we recommend that PET researchers publish their voxel sizes and reconstruction details to allow for later comparison with subsequent data.

The harmonization technique presented here is easy to implement for comparing and combining data; however, the technique has limitations. (1) It is limited by the scanner with the worst resolution. That is, the technique can only make an image with better resolution look like an image with poorer resolution. It is not possible to use the technique to improve a lower resolution dataset. (2) We derived the optimal kernel on a static phantom, but the contrast—and thus the spill-out/spill-in—changes continually in dynamic scans. Future studies should explore *dynamic* phantoms for the possibility of developing time frame-specific harmonization. (3) Finally, we used a phantom with a single compartment. All cavities in the phantom contained the same radioactivity concentration. Researchers may want to consider phantoms with separate fillable compartments, such as those used by Fahey et al. [17] and Mannheim et al. [18] to assess inter-scanner variability.

In conclusion, we developed a method to harmonize HRRT and HR+ [^11^C]raclopride images and validated the method using images of human subjects on both scanners. The results allow for [^11^C]raclopride striatal regional BP_ND_ estimates from both scanners to be compared and/or combined.

## Supplementary Information

**Additional file 1 Fig. S1**. The sum of squared errors (SSE) for various isotropic gaussian filters with FWHM ranging from 0 to 10 mm. The minimum SSE occurred at FWHM= 4.5mm. Note that the SSE is constant for FWHM values less than the smallest dimension of the HR+ voxel size (2.06 mm).

## Data Availability

The data used in this study are not openly available at this time because the study is ongoing. Software and code are available upon request.

## Abbreviations

BP_ND_: Non-displaceable binding potential
ADNI: Alzheimer's Disease Neuroimaging Initiative
FWHM: Full width at half maximum
OSEM: Ordered subset expectation maximum
hHRRT: Harmonized HRRT
SSE: Sum of squared errors
SRTM: Simplified reference tissue model
PD: Percent difference
T/RT: Test–retest
