# Supplementary material for: Harmonization of [11C]raclopride brain PET images from the HR+ and HRRT: method development and validation in human subjects
Source: EJNMMI Phys. 2022 Apr 13;9:27. doi: 10.1186/s40658-022-00457-z (PMC9008103; doi:10.1186/s40658-022-00457-z)
Supplement: Supplementary file 1 — Additional file 1 Fig. S1. The sum of squared errors (SSE) for various isotropic gaussian filters with FWHM ranging from 0 to 10 mm. The minimum SSE occurred at FWHM= 4.5mm. Note that the SSE is constant for FWHM values less than the smallest dimension of the HR+ voxel size (2.06 mm). [file 40658_2022_457_MOESM1_ESM.docx]

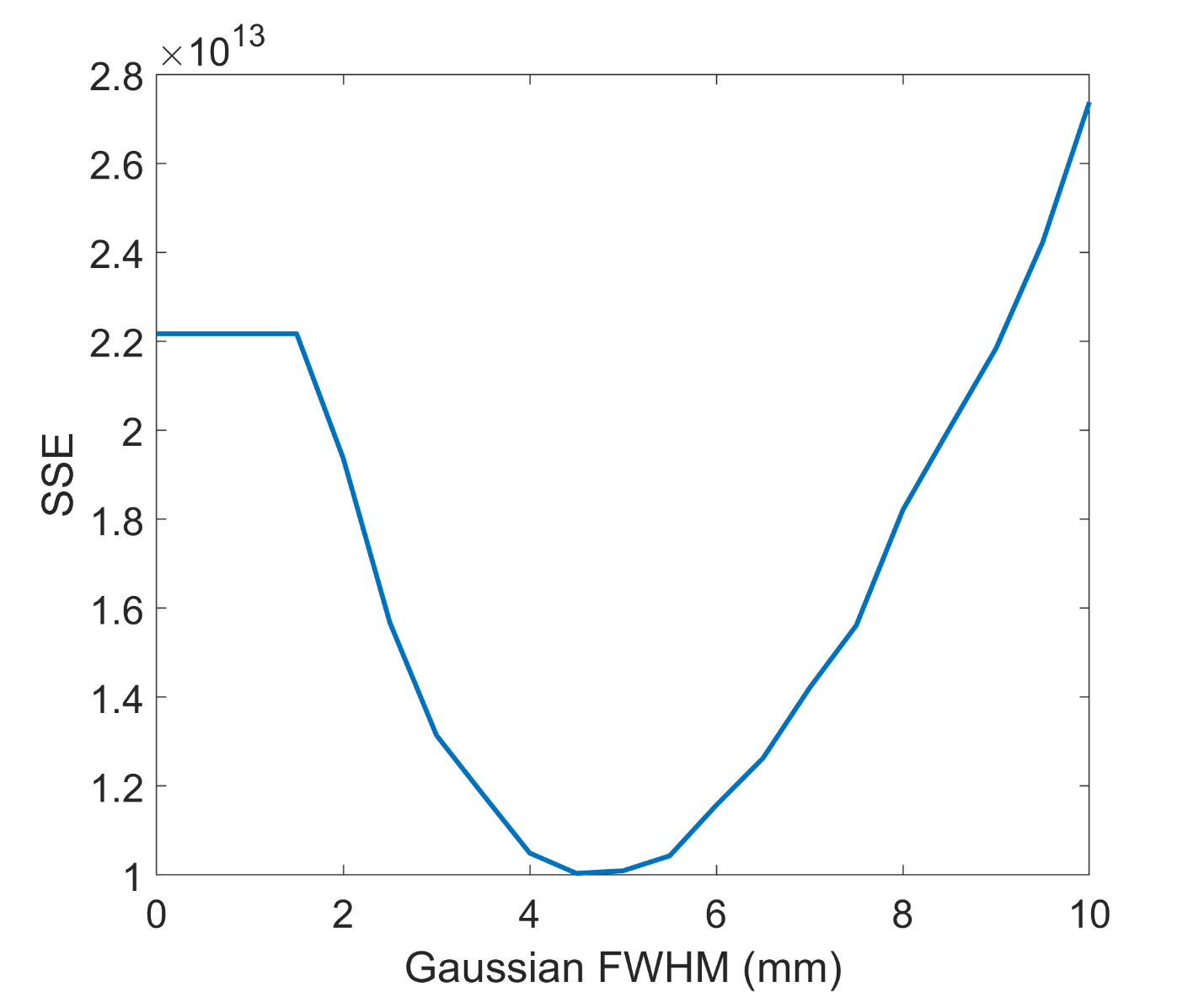


Figure S1. The sum of squared errors (SSE) for various isotropic gaussian filters with FWHM ranging from 0 to 10 mm. The minimum SSE occurred at FWHM= 4.5mm. Note that the SSE is constant for FWHM values less than the smallest dimension of the HR+ voxel size (2.06 mm).
